# Supplementary material for: Recovery from acute SARS-CoV-2 infection and development of anamnestic immune responses in T cell-depleted rhesus macaques
Source: bioRxiv. 2021 Apr 4:2021.04.02.438262. Preprint. [Version 1] doi: 10.1101/2021.04.02.438262 (PMC8020972; doi:10.1101/2021.04.02.438262)

bioRxiv preprint doi: https://doi.org/10.1101/2021.04.02.438262; this version posted April 4, 2021. The copyright holder for this preprint (which was not certified by peer review) is the author/funder. This article is a US Government work. It is not subject to copyright under 17 USC 105 and is also made available for use under a CC0 license.

Supplementary data Fig. 1a. Ki-67 staining of CD8+ T cells

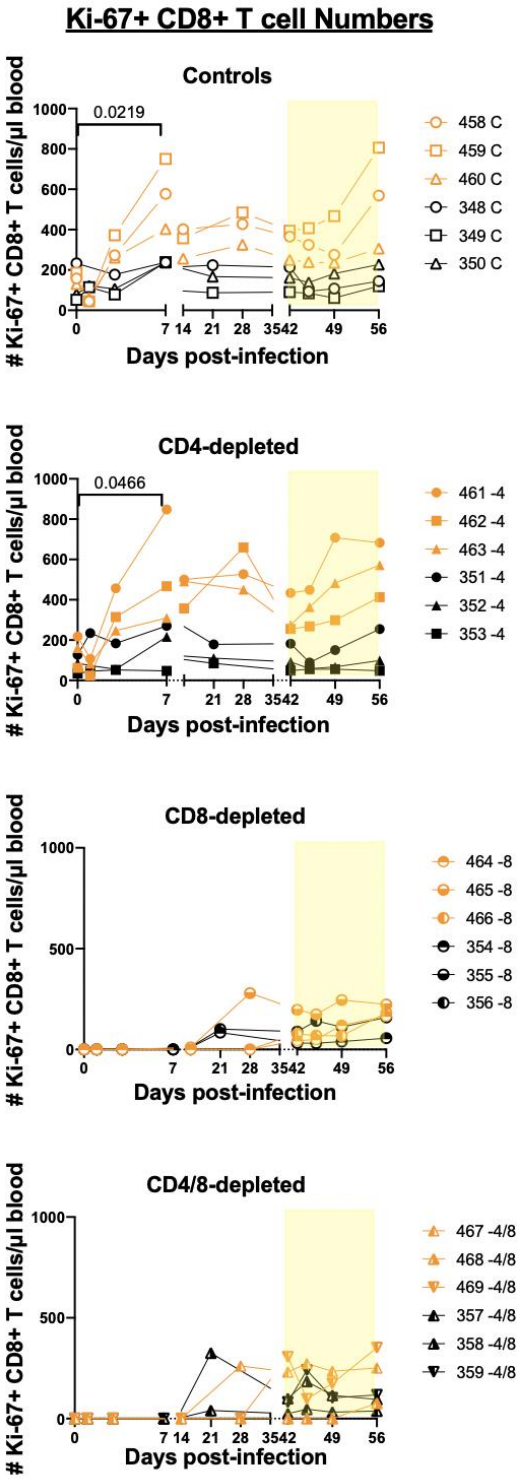

bioRxiv preprint doi: https://doi.org/10.1101/2021.04.02.438262; this version posted April 4, 2021. The copyright holder for this preprint (which was not certified by peer review) is the author/funder. This article is a US Government work. It is not subject to copyright under 17 USC 105 and is also made available for use under a CC0 license.

Supplementary data Fig. 1b. Gating strategies for Flow Cytometry.

a. Gating Strategy for CD4+ T cells

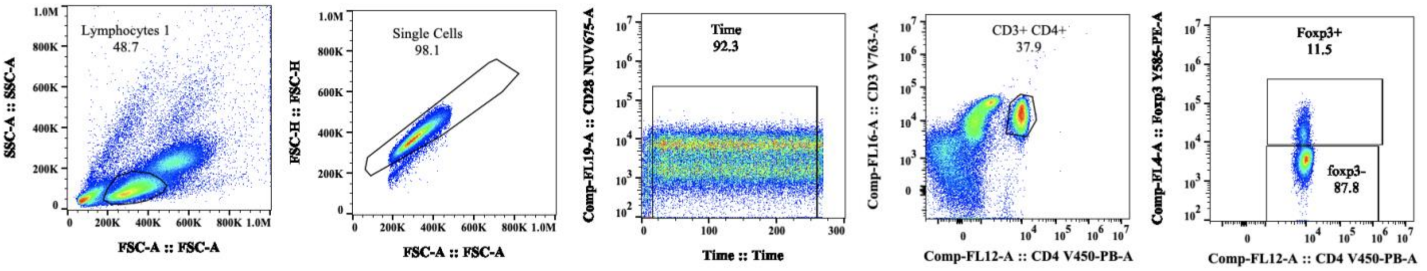

b. Gating Strategy for CD8+ T cells

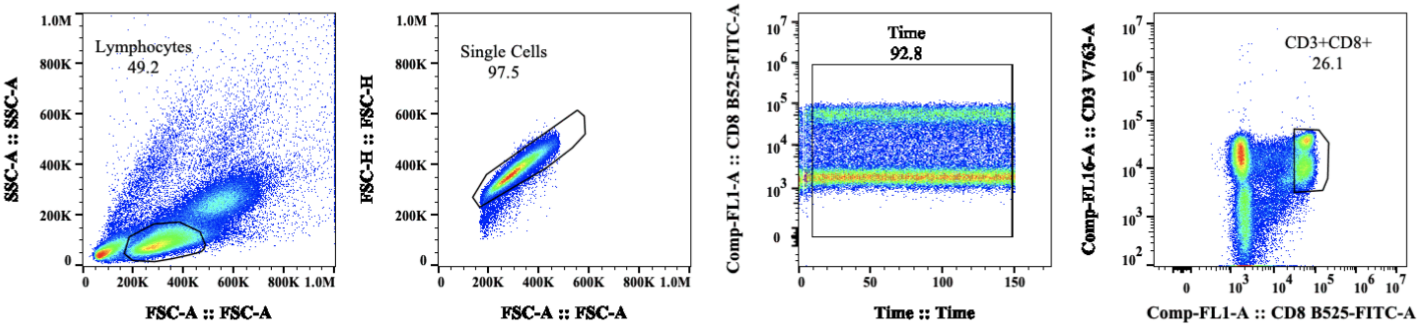

c. Gating strategy for B cells

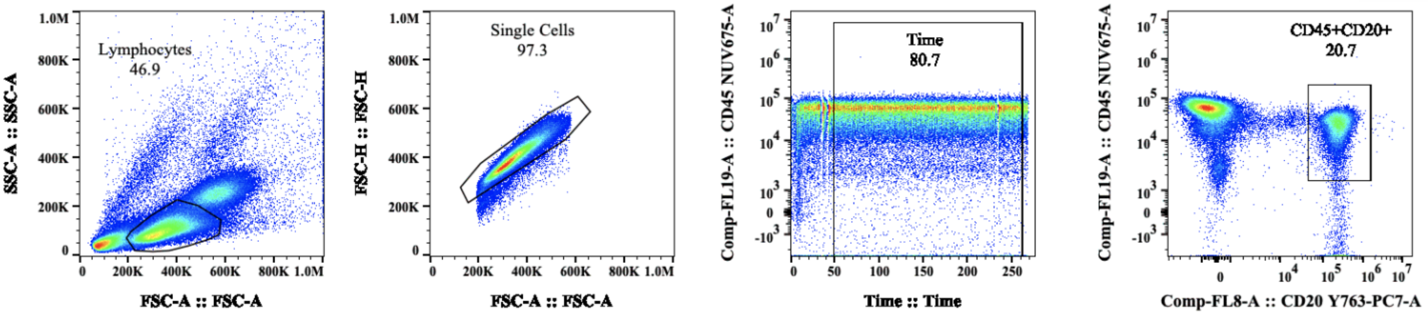

bioRxiv preprint doi: https://doi.org/10.1101/2021.04.02.438262; this version posted April 4, 2021. The copyright holder for this preprint (which was not certified by peer review) is the author/funder. This article is a US Government work. It is not subject to copyright under 17 USC 105 and is also made available for use under a CC0 license.

Supplementary data Fig. 3

Clinical data n- CoV 2019 CoV 348-359

Animal ID	Day 0 -13	Day 42-56	Necropsy notes
**C-1**	Hunched posture, ruffled fur, pale appearance, increased irregular respirations<br>Score:3/5/5/10/10/10/10/10/10/5/5/5/5/5/0 (98)	Reduced appetite<br>Score:<br>3/0/0/0/0/0/0/0/0/0/0/0/0/0/0 (3)	normal
**C-2**	Reduced appetite, abdominal breathing, pale appearance<br>Score :3/5/3/3/3/3/5/3/3/3/3/3/3/3/0 (46)	Reduced appetite<br><br>Score: 3/3/3/3/3/0/0/0/0/0/0/0/0/0 (15)	normal
**C-3**	Reduced appetite, slightly irregular respirations<br>Score: 3/3/8/3/3/5/5/8/3/0/3/3/0/3/0 (50)	Reduced appetite<br><br>Score:3/3/3/0/3/0/0/0/0/0/0/0/0/0 (12)	Right lung: adhesi[illegible]
**CD4-1**	Reduced appetite, increased abdominal respirations, pale appearance<br>Score: 0/8/8/8/8/8/5/5/8/5/5/0/2/2/0 (72)	Reduced appetite, slightly irregular respirations<br>Score: 0/3/2/2/2/0/0/0/0/0/0/0/0/0 (9)	Lung FTC (BAL)
**CD4-2**	Reduced appetite<br>Score: 3/3/3/3/3/3/3/3/0/3/3/3/0/0 (36)	Reduced appetite<br>Score:3/3/3/3/3/0/0/0/0/0/0/0/0/0<br>(12)	normal
**CD4-3**	Reduced appetite, increased irregular respirations<br>Score:0 /8/8/10/8/3/5/5/3/3/0/3/0/2/0 (58)	Reduced appetite, slightly irregular respirations<br>Score:0/5/5/2/5/0/0/0/0/0/0/0/0/0<br>(17)	normal
**CD8-1**	Reduced appetite, increased abdominal respirations<br>Score:0/8/10/8/8/8/8/8/8/3/3/3/0/0 (75)	Reduced appetite<br>Score: 0/3/3/0/0/0/0/0/0/0/0/0/3/0/0<br>(9)	normal
**CD8-2**	Reduced appetite, increased abdominal respirations<br>Score:3/8/8/8/5/3/0/3/0/0/3/3/0/0/0 (44)	Reduced appetite<br><br>Score: 3/3/0/0/0/0/0/0/0/0/0/0/0/0<br>(6)	Mediastinal LN e[illegible]
**CD8-3**	Reduced appetite, slightly irregular respirations<br>Score: 0/3/2/5/2/2/2/0/3/0/0/0/0/0/0 (19)	Reduced appetite<br>Score:0/3/3/0/0/0/0/0/0/0/0/0/0/0<br>(6)	normal
**CD4/8-1**	Slightly irregular abdominal respirations<br>Score: 0/0/0/0/0/2/2/2/2/0/2/0/0/0/0 (10)	Reduced appetite<br>Score:0/0/3/0/0/0/0/0/0/0/0/0/0/0<br>(3)	normal
**CD4/8-2**	Reduced appetite, hunched posture, ruffled fur, increased irregular abdominal respirations, nasal discharge<br>Score: 3/3/8/13/13/13/13/8/8/5/5/5/2/2/0<br>(103)	Reduced appetite, increased abdominal respirations<br>Score: 0/3/2/2/8/0/0/0/2/0/0/0/0/0<br>(17)	normal

bioRxiv preprint doi: https://doi.org/10.1101/2021.04.02.438262; this version posted April 4, 2021. The copyright holder for this preprint (which was not certified by peer review) is the author/funder. This article is a US Government work. It is not subject to copyright under 17 USC 105 and is also made available for use under a CC0 license.

**CD4/8-3**	Reduced appetite, increased irregular respirations, pale appearance<br>Score: 3/8/7/8/8/5/5/8/8/5/5/3/0/0/0/0 (73)	Reduced appetite<br><br>Score: 0/3/0/0/0/0/0/0/0/0/0/0/0/0 (3)	normal

Clinical data n- CoV 2019 CoV 458-469

**Animal ID**	Day 0 -14	Day 42-46	Necropsy notes
**C-4**	Ruffled fur, slightly increased irregular respirations<br>Score: 0/0/0/5/10/5/0/0/0/0/0/0/0/0/0 (20)	Slightly irregular respirations<br><br>Score:3/3/3/3 (12)	normal
**C-5**	Reduced appetite, abdominal slightly irregular breathing<br>Score :0/6/8/6/3/8/6/3/0/5/0/0/3/5/3 (56)	Reduced appetite, slightly irregular respirations<br>Score:0/0/6/0 (6)	normal
**C-6**	Normal<br>Score: 0/0/0/0/0/0/0/0/3/3/3/5/0/0 (14)	Normal<br>Score:0/0/0/0 (0)	normal
**CD4-4**	normal<br>Score: 0/0/0/0/0/0/0/0/3/3/0/0/0/0/0 (6)	normal<br>Score: 0/0/0/0 (0)	normal
**CD4-5**	Reduced appetite<br>Score: 0/3/5/5/3/0/5/3/0/0/5/0/0/3/0 (32)	normal<br>Score:0/0/0/0 (0)	normal
**CD4-6**	normal<br>Score:0 /0/0/0/0/0/0/0/0/0/5/0/0/0/0 (5)	normal<br>Score:0/0/0/0 (0)	normal
**CD8-4**	Reduced appetite, increased irregular abdominal respirations<br>Score:0/3/8/6/6/3/8/5/3/5/3/5/3/0/0 (58)	Normal<br><br>Score: 0/0/0/0 (0)	normal
**CD8-5**	Reduced appetite, increased abdominal respirations<br>Score:0/3/8/8/3/3/0/0/0/3/0/0/3/0/0 (31)	normal<br><br>Score: 0/0/0/0 (0)	normal
**CD8-6**	Reduced appetite, increased respirations<br>Score: 0/3/0/5/5/0/0/0/0/0/3/0/3/0/0 (19)	Reduced appetite<br>Score:0/3/0/0 (3)	normal
**CD4/8-4**	increased abdominal respirations, ruffled fur, reduced appetite<br>Score: 0/5/5/15/5/5/0/0/0/0/0/0/0/0/0 (30)	normal<br>Score:0/0/0/0 (0)	normal
**CD4/8-5**	Reduced appetite, ruffled fur, increased irregular abdominal respirations<br>Score: 0/8/8/10/5/8/10/10/3/5/5/5/3/0/0 (80)	Normal<br><br>Score: 0/0/0/0 (0)	normal
**CD4/8-6**	Reduced appetite, increased irregular respirations, pale appearance<br>Score: 0/6/5/15/13/13/13/15/6/3/5/5/3/0/0 (102)	normal<br><br>Score: 0/0/0/0	normal

bioRxiv preprint doi: https://doi.org/10.1101/2021.04.02.438262; this version posted April 4, 2021. The copyright holder for this preprint (which was not certified by peer review) is the author/funder. This article is a US Government work. It is not subject to copyright under 17 USC 105 and is also made available for use under a CC0 license.

Supplementary data Fig. 4

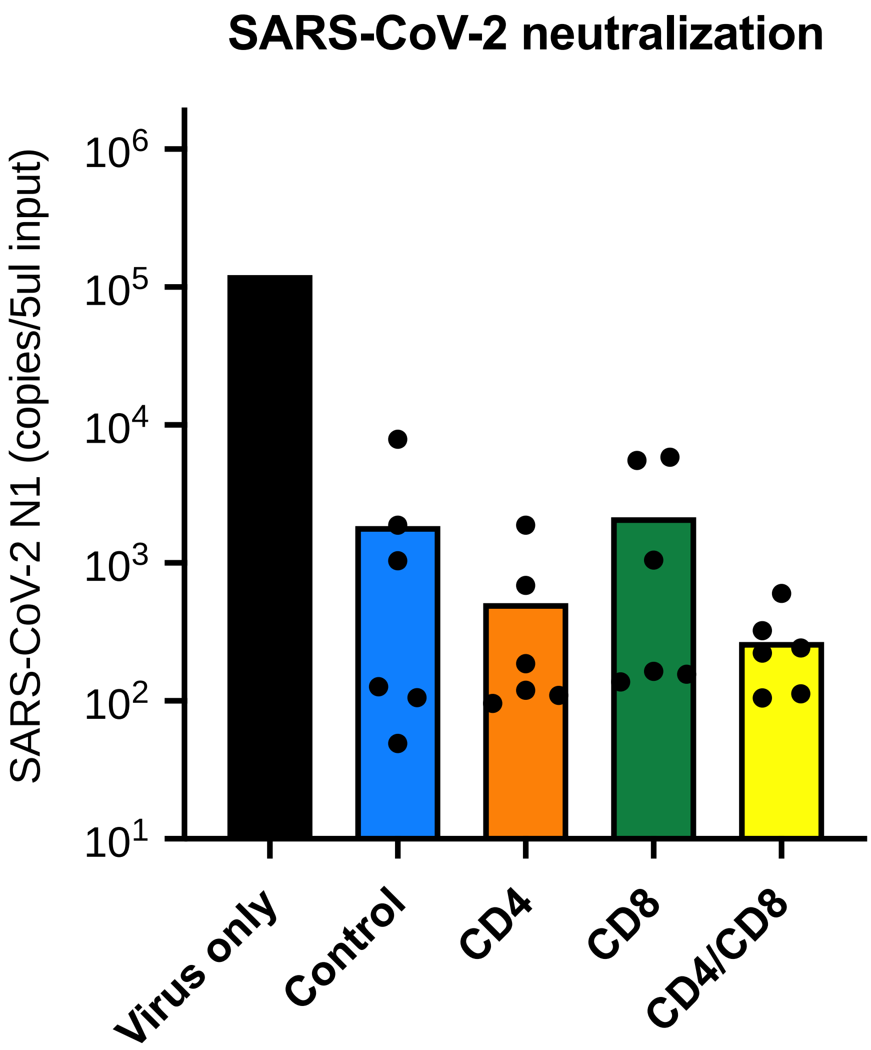

SARS-CoV-2 neutralization assay with live coronoavirus. Day 28 post-infection sera from all NHP were diluted 1:20 and tested for neutralization of SARS-CoV-2. Each dot represents the result from an individual macaque.

bioRxiv preprint doi: https://doi.org/10.1101/2021.04.02.438262; this version posted April 4, 2021. The copyright holder for this preprint (which was not certified by peer review) is the author/funder. This article is a US Government work. It is not subject to copyright under 17 USC 105 and is also made available for use under a CC0 license.

Supplementary data Fig. 5a. CD4+ T cell staining in spleens.

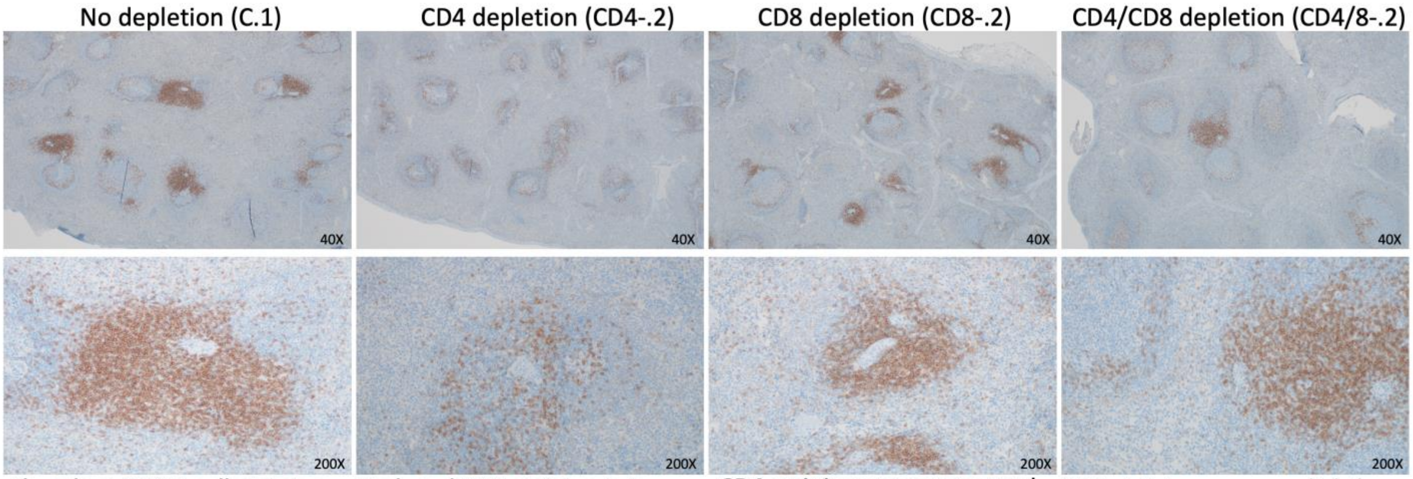

Abundant CD4 T cell staining in periarteriolar lymphoid sheaths and the mantle zone of lymphoid follicles. Normal.

Reduced CD4 staining is in periarteriolar lymphoid sheaths and possibly the mantle zone of lymphoid follicles.

CD4 staining appears normal.

CD4 staining appears slightly reduced.

Supplementary data Fig. 5b. CD8+ T cells in spleen

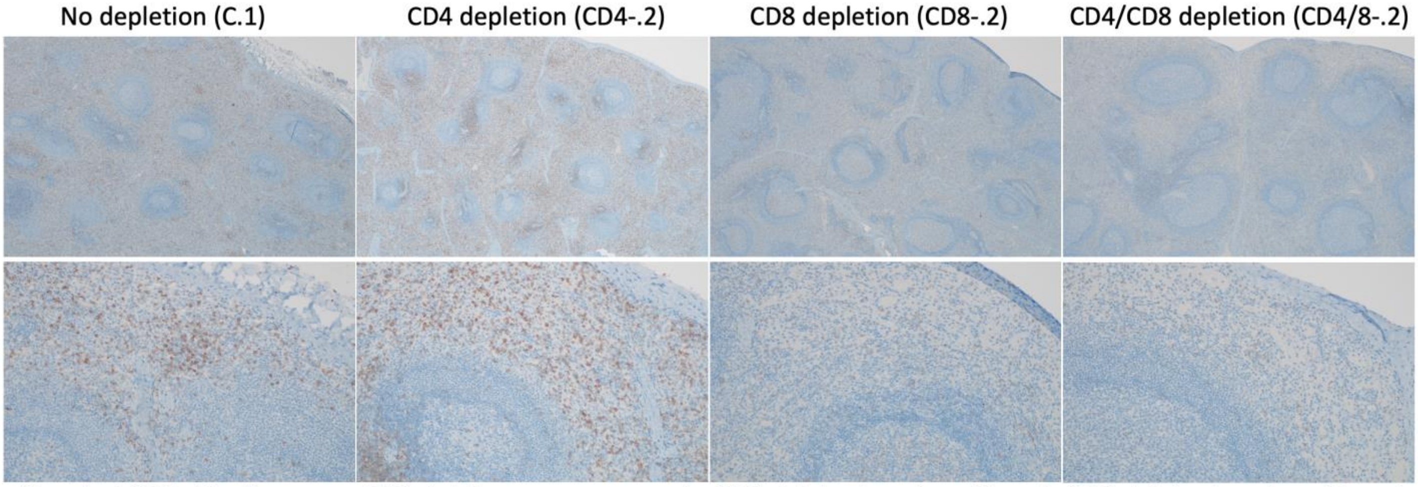

Abundant CD8+ T cells within the red pulp between lymphoid follicles. Normal.

CD8 staining appears normal.

CD8 staining is reduced in red pulp.

CD8 staining is reduced in red pulp.

bioRxiv preprint doi: https://doi.org/10.1101/2021.04.02.438262; this version posted April 4, 2021. The copyright holder for this preprint (which was not certified by peer review) is the author/funder. This article is a US Government work. It is not subject to copyright under 17 USC 105 and is also made available for use under a CC0 license.

Supplemetary Figure 6. IP-10 levels in broncho-alveolar lavage fluids.

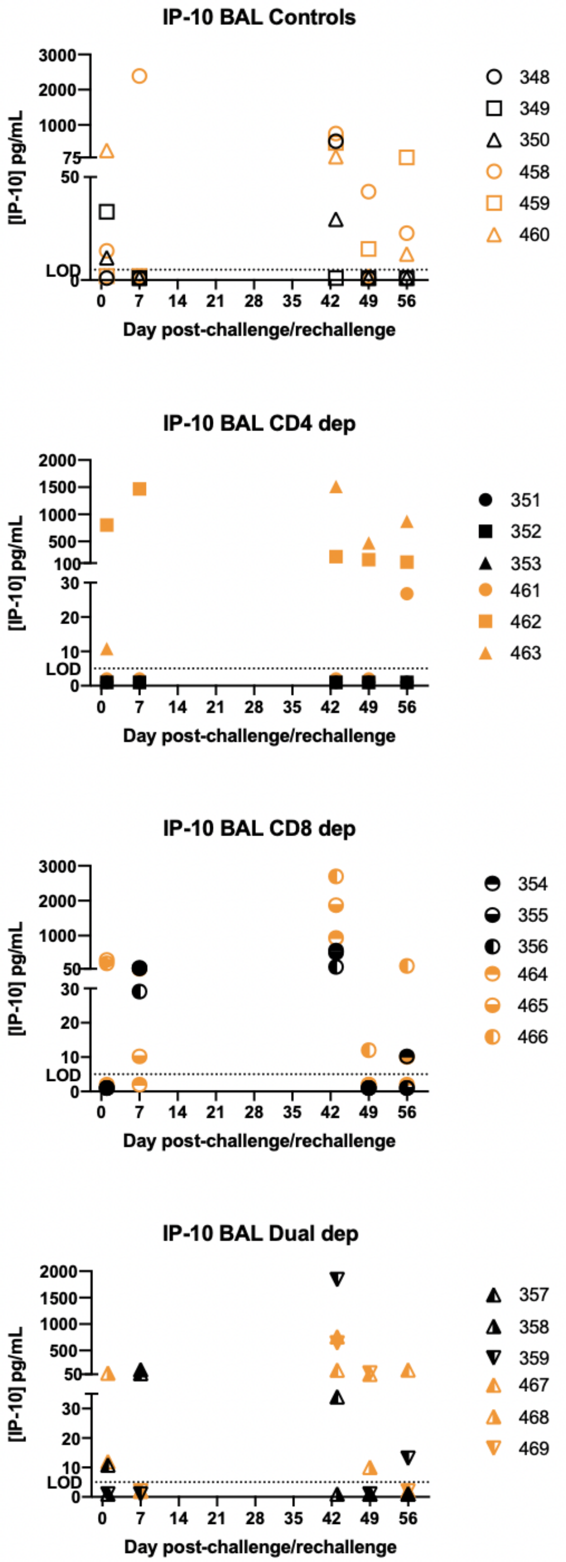

Supplement: 1 [file NIHPP2021.04.02.438262-supplement-1.pdf]
